# A Novel Natural Chromogenic Visual and Luminescent Sensor Platform for Multi-Target Analysis in Strawberries and Shape Memory Applications

**DOI:** 10.3390/foods14162791

**Published:** 2025-08-11

**Authors:** Hebat-Allah S. Tohamy

**Affiliations:** Cellulose and Paper Department, National Research Centre, 33 El Bohouth Str., Dokki, Giza P.O. Box 12622, Egypt; hebasarhan89@yahoo.com

**Keywords:** shape memory, betalains, beet root carbon dots, natural sensor, food packaging, heavy meatal sensor, chromogenic sensor, luminescent sensor, pH-sensing, visual detection

## Abstract

Carboxymethyl cellulose (CMC) films, derived from sugarcane bagasse agricultural waste (SCB) incorporated with Betalains-nitrogen-doped carbon dots (Betalains-N–CQDs), derived from beet root waste (BR), offer a sustainable, smart and naked-eye sensor for strawberry packaging due to their excellent fluorescent and shape memory properties. These CMC-Betalains-N–CQDs aim to enhance strawberry preservation and safety by enabling visual detection of common food contaminants such as bacteria, fungi and Pb(II). Crucially, the CMC-Betalains-N–CQD film also exhibits excellent shape memory properties, capable of fixing various shapes under alkaline conditions and recovering its original form in acidic environments, thereby offering enhanced physical protection for delicate produce like strawberries. Optical studies reveal the Betalains-N–CQDs’ pH-responsive fluorescence, with distinct emission patterns observed across various pH levels, highlighting their potential for sensing applications. Scanning Electron Microscopy (SEM) confirms the successful incorporation of Betalains-N–CQDs into the CMC matrix, revealing larger pores in the composite film that facilitate better interaction with analytes such as bacteria. Crucially, the CMC-Betalains-N–CQD film demonstrates significant antibacterial activity against common foodborne pathogens like Escherichia coli, Staphylococcus aureus, and Candida albicans, as evidenced by inhibition zones and supported by molecular docking simulations showing strong binding interactions with bacterial proteins. Furthermore, the film functions as a fluorescent sensor, exhibiting distinct color changes upon contact with different microorganisms and Pb(II) heavy metals, enabling rapid, naked-eye detection. The film also acts as a pH sensor, displaying color shifts (brown in alkaline, yellow in acidic) due to the betalains, useful for monitoring food spoilage. This research presents a promising, sustainable, and multifunctional intelligent packaging solution for enhanced food safety and extended shelf life.

## 1. Introduction

The inherent vulnerability of fresh produce, particularly strawberries, to contamination by heavy metals like lead (Pb(II)) and pathogenic bacteria poses a significant public health concern and necessitates robust safety monitoring [1]. While highly accurate, conventional detection methods for these contaminants, such as atomic absorption spectroscopy, are often laboratory-bound, time-consuming, and require specialized equipment, rendering them impractical for rapid, on-site assessment crucial for perishable goods [2,3,4]. To overcome these limitations and ensure real-time food safety, there is a growing demand for innovative, accessible sensing technologies. In response, this research leverages the exceptional properties of carboxymethyl cellulose (CMC) as a biocompatible, film-forming matrix, combined with novel carbon quantum dots (CQDs) derived from sustainable agricultural waste like beet root peels (BR). These colorful CQDs, with their inherent fluorescence, antibacterial activity, and strong adsorption capabilities, are integrated into the CMC polymer network to fabricate advanced nanocomposite films. The inherent biocompatibility, non-toxic nature, biodegradability, and renewability of natural polymers, including polysaccharides, have led to their extensive use in film production [5,6,7,8,9]. Within the polysaccharide category, carboxymethyl cellulose (CMC) is particularly favored in research and industrial applications due to its superior structural and functional attributes, rendering it well-suited for food packaging [10,11,12,13,14]. For instance, the presence of carboxymethyl groups along the cellulose backbone imparts enhanced water solubility and film-forming capabilities compared to unmodified cellulose [15,16,17,18,19,20]. This allows for the creation of smooth, uniform films that can act as effective barriers [21,22,23]. Furthermore, the hydroxyl and carboxylate groups on CMC offer opportunities for chemical modification and crosslinking, enabling the tailoring of film properties such as mechanical strength, flexibility, and permeability to suit specific food packaging requirements [6,24,25]. Its biodegradability also aligns with growing environmental concerns regarding plastic waste, making CMC a sustainable alternative for various packaging applications. The versatility and tunability of CMC, therefore, contribute significantly to its suitability and popularity in the realm of food packaging [26,27,28]. Nanocomposite materials, created by integrating active nanomaterials within a polymer matrix, offer enhancements in antimicrobial efficiency [29,30,31,32,33,34,35]. The interaction between nanoparticles and the polymer network, occurring via either covalent linkages or weaker intermolecular forces, plays a crucial role in controlling the release and migration of molecules [36,37,38,39,40,41].

Carbon quantum dots (CQDs), characterized by their nanoscale dimensions (typically below 10 nm), present themselves as compelling reinforcing agents due to their favorable attributes, including high water solubility, biocompatibility, low toxicity, and sustainable sourcing [34,35,42]. The presence of hydroxyl, amino, and carboxyl functional groups on their surface endows them with fluorescence and antibacterial activity, rendering them highly suitable for food preservation applications [43,44,45,46,47]. Furthermore, the strong adsorption of CQDs onto biological surfaces expands their potential utility [48,49,50]. While agricultural waste streams like BR have been successfully utilized to derive CQDs for incorporation into active green packaging, BR, despite its abundance of bioactive compounds such as betalains, which are water-soluble pigments that function as the primary source of color [51]. The natural color diversity within betalains allows for straightforward colorimetric detection by the naked eye, directly reflecting alterations in the chemical surroundings or the presence of specific analytes. As a result, the color of the synthesized CQDs originating from these betalains can be visually evaluated, providing a simplified method for rapid, on-site analysis. Furthermore, these BR-derived CQDs present a safer and more environmentally friendly option, exhibiting fluorescence that is observable without sophisticated equipment.

The accumulation of agrowastes, the residues generated from agricultural activities, presents a significant global challenge with far-reaching environmental, economic, and social implications. These wastes, encompassing crop residues (straw, stalks, leaves), livestock manure, agro-industrial byproducts, and even discarded packaging materials, are produced in massive quantities annually due to increasing agricultural production to feed a growing global population. Utilizing sugarcane bagasse (SCB) for carboxymethyl cellulose (CMC) production offers a dual advantage: it not only provides a sustainable alternative to traditional cellulose sources but also addresses the issue of SCB accumulation. Instead of being underutilized or burned (which leads to environmental pollution), SCB, an abundant agricultural waste product, can be transformed into a valuable material. This approach supports a circular economy by converting waste into a resource, reducing disposal challenges, and minimizing the environmental footprint associated with SCB buildup [52,53,54]. The same for CQDs, the current research investigates the colorful CQDs from BR wastes, thereby promoting the valorization of agricultural waste and aligning with global sustainability initiatives. Importantly, these CQD-based nanocomposites hold promise for the advancement of active green packaging solutions aimed at enhancing food preservation and extending the shelf life of perishable goods.

Ensuring food safety is a critical global concern, primarily due to the potential contamination of both processed and fresh produce with heavy metals and pathogenic bacteria. Heavy metals, including lead, mercury, and cadmium, can accumulate in food through environmental pollution, posing significant health risks to consumers [55]. Similarly, pathogenic bacteria, such as *Salmonella*, *E. coli*, and *S. aureus*, can contaminate food at various stages of the supply chain, leading to foodborne illnesses [56,57]. The presence of these contaminants necessitates stringent food safety measures to protect public health and maintain consumer confidence in the food supply. Among heavy metals, lead (Pb(II)) poses a particularly insidious threat due to its widespread environmental presence and severe toxic effects on human health [1]. Lead can enter the food supply through various routes, including contaminated soil and water in agricultural areas, industrial pollution, and even through certain food packaging materials or traditional cookware [58,59]. Once ingested, Pb(II) accumulates in the body over time, affecting multiple organ systems. It is especially detrimental to children, where even low levels of exposure can lead to irreversible neurological damage, including developmental delays, reduced IQ, learning difficulties, and behavioral problems [60,61]. In adults, chronic lead exposure is linked to serious health issues such as increased blood pressure, cardiovascular problems, kidney damage, reproductive issues, and neurological disorders like memory loss and difficulties with concentration [62]. Methods like atomic absorption spectroscopy are highly accurate for quantifying lead levels, but they are typically time-consuming, require specialized equipment and expertise, and are not practical for rapid, on-site testing [63,64]. This limitation highlights the need for new sensing technologies that are simpler, more sensitive, and capable of providing real-time detection [42]. Strawberries, like other fresh produce, are susceptible to contamination by both heavy metals and pathogenic bacteria [65,66]. They can absorb heavy metals, such as Pb(II), from contaminated soil or water, with contamination occurring due to industrial pollution, agricultural practices, or proximity to contaminated sites [67]. Strawberries can also be contaminated with pathogens, including bacteria like *Candida*, *E. coli*, and *S. aureus* at various stages of the supply chain [66,67,68]. Factors contributing to pathogen contamination include contact with contaminated soil or water, the use of contaminated compost, poor hygiene practices, contact with contaminated equipment, and cross-contamination during storage or transportation [69]. The consumption of contaminated strawberries can lead to adverse health effects, necessitating effective food safety measures. Visually identifying chromium and bacteria presents a notable benefit due to its simplicity and ease of use, particularly for testing in the field. The film’s color change and fluorescent behavior allow for a quick and uncomplicated way to evaluate the safety of Strawberry samples. This synergistic combination enables the development of a smart packaging or sensing system capable of providing rapid, visually identifiable detection of contaminants, thereby offering a practical and environmentally friendly solution to enhance the safety and extend the shelf life of strawberries and other perishable foods. This study introduces a significant advancement in sensing technology by presenting, for the first time, a naked-eye fluorescent sensor derived from beetroot waste. This pioneering approach leverages the inherent properties of naturally occurring compounds within beetroot to synthesize CQDs that exhibit remarkable fluorescence, observable without sophisticated equipment. The novelty of this work lies in the sustainable and cost-effective preparation of these highly functional CQDs from an abundant agricultural byproduct, which are then integrated into a sensor designed for simplistic, rapid, and visual detection. This innovative methodology not only offers a green alternative to traditional sensor fabrication but also paves the way for accessible, on-site monitoring solutions with direct visual feedback, representing a notable leap in the development of environmentally conscious and user-friendly sensing platforms.

## 2. Materials and Methods

### 2.1. Materials

Nitrogen-doped carbon dots (Betalains-N–CQDs) were synthesized using beet root (BR) obtained from an Egyptian market in the local area. While SCB was obtained from the Quena factory in Egypt after SCB treatment to produce molasses. The monochloro-acetic acid and urea were purchased from Sigma-Aldrich (St. Louis, MO, USA). All materials were used as received without further purification.

### 2.2. Preparation of Nitrogen-Doped Carbon Quantum Dots (Betalains-N–CQDs)

BR served as the precursor for generating nitrogen-doped carbon quantum dots (Betalains-N–CQDs) via a multi-step method. This process began with the creation of a consistent combination of 4 g BR, 9.33 g NaOH, 9.33 g urea, and 100 mL water, which was then subjected to sequential freezing, sonication, and microwave irradiation at 700 W for 7 min.

### 2.3. Preparation of Cellulose

A multi-stage extraction process was employed to obtain purified α-cellulose, starting with 150 g of SCB. First, the SCB underwent prehydrolysis in an autoclave using dilute hydrochloric acid at 120 °C for a 2-h duration. Subsequently, an alkaline treatment was performed on the pretreated material using a solution of 20 g of sodium hydroxide in 300 mL of water, with heating at 170 °C for 2 h, resulting in a brownish, fibrous pulp. To eliminate lignin, 80 g of this pulp was bleached with a 3% HClO_2_ solution (prepared by dissolving 2.4 g in 4750 mL of water) under acetic acid conditions at 80 °C for 2 h, while maintaining the pH between 1 and 3 through controlled additions of acetic acid [54,70].

### 2.4. Preparation of Carboxymethyl Cellulose (CMC)

The synthesis of carboxymethyl cellulose (CMC) involved reacting 15 g of cellulose with a 30% sodium hydroxide solution, followed by the addition of 18 g of monochloroacetic acid. This reaction mixture was then heated using microwave irradiation until the cellulose fully dissolved. Subsequently, the CMC product was precipitated by the addition of 70% ethanol, separated through filtration, and then dried in an oven [2].

### 2.5. Preparation of Nitrogen-Doped Carbon Quantum Dots-Carboxymethyl Cellulose Hydrogel Film (CMC-Betalains-N–CQDs)

The preparation of a composite film involving Betalains-N–CQDs and CMC was carried out using a solution casting technique on a Teflon plate. In detail, 1 g of CMC was dissolved in 50 mL of deionized water. For the Betalains-N–CQD-containing film, 15% (wt/wt) of Betalains-N–CQDs was incorporated into the CMC solution, and the resulting film was designated as CMC-Betalains-N–CQDs. A control film, labeled CMC film, was produced following an identical procedure but without the inclusion of Betalains-N–CQDs.

### 2.6. Nitrogen-Doped Carbon Quantum Dots-Carboxymethyl Cellulose Hydrogel Film Application to Monitor and Preserve Lead, Bacteria and Fungi in Strawberries

An experiment was conducted using fresh strawberries obtained from a local market in Cairo, Egypt. To evaluate the Pb(II) detection capability of the films, strawberries were prepared with a Pb(II) concentration of 3.11 mg/kg, a level comparable to that found in contaminated canned products [71]. These prepared strawberries were then used in sensing experiments. Two strawberry groups were individually placed in containers, each enclosed with CMC-Betalains-N–CQD film to detect bacteria, fungi and yeast (strawberries without Pb(II) treatment) and Pb(II) (strawberries with Pb(II) treatment). The containers were stored in a refrigerator, and a smartphone camera was used to monitor and record the color changes of the strawberries over this period.

### 2.7. Shape Memory Test

To test the shape memory of CMC film and CMC-Betalains-N–CQD composite films (specifically those with 15 wt% Betalains-N–CQDs), we first made them flexible by soaking them in a 0.5 M HCl solution. For a qualitative look at their shape memory, we bent the softened films into new shapes, held those shapes by putting them in a 0.5 M NaOH solution, and then used a 0.5 M HCl solution to make them return to their original form. For a quantitative measurement, we determined the shape fixity ratio (R_f_) by bending a film strip into a U-shape and keeping it that way in a 0.5 M NaOH solution for a set time. We then immersed it in a 0.5 M HCl solution to measure the shape recovery ratio (R_r_). These ratios were calculated using specific equations:

(1) $$R_{f}\;=\;\frac{\theta_{t}}{\theta_{i}}\;\times \;100\%$$

(2) $$R_{r}=\frac{\left(\theta_{i}-\theta_{f}\right)}{\theta_{i}}\;\times \;100\%$$

where, θ_i_, θ_t_, and θ_f_ are the given angle, temporarily fixed angle and terminal angle, respectively [72].

### 2.8. Instruments

The pH sensitivity of the film was evaluated by immersing it in acidic and alkaline buffer solutions for 1 min, adhering to the method described by Tohamy, visual color alterations were recorded using a smartphone [36]. For fluorescent study, a Jasco FP-6500 spectrofluorometer (Jasco Intern. Co., Tokyo, Japan) equipped with a 150-watt xenon lamp, featuring a 150-watt xenon arc lamp, was used to perform fluorescence measurements. To analyze the functional groups, Fourier Transform Infrared (FTIR) spectroscopy was carried out using a Mattson 5000 spectrometer (Unicam, UK). Samples were prepared using the KBr pellet technique. The average hydrogen bond strength (MHBS) was then calculated based on Equation (3).

(3) $$\mathrm{MHBS}=\frac{A_{OH}}{A_{CH}}$$

where A_OH_ and A_CH_ refer to the FTIR absorbance of the OH and CH peaks, respectively [44]. The density functional theory (DFT) calculations were performed using the Gaussian 6.0. 16 09 W software package. Geometry optimization was carried out using the Berny algorithm. This computational approach enabled the investigation of parameters such as optimized molecular geometries and their respective ground state energies. The total energy (E_T_), the energy of the highest occupied MO E_HOMO_, the energy of the lowest unoccupied MO E_LUMO_, the energy gap (E_g_), the dipole moment (μ), the absolute hardness (η), the absolute softness (σ), the chemical softness (S), and the additional electronic charge (ΔN_max_) were calculated [36,73].

(4) $$E_{gap}=(E_{LUMO}-E_{HOMO})$$

(5) $$\eta =\frac{(E_{LUMO}+\;E_{HOMO})\;\;}{2}$$

(6) $$\sigma =\frac{1\;\;}{\eta }$$

(7) $$S=\frac{1\;\;}{2\eta }$$

(8) $$\Delta N_{max}=\frac{-Pi\;\;}{\eta }$$

## 3. Results and Discussion

### 3.1. Optical Study

**Figure 1:**
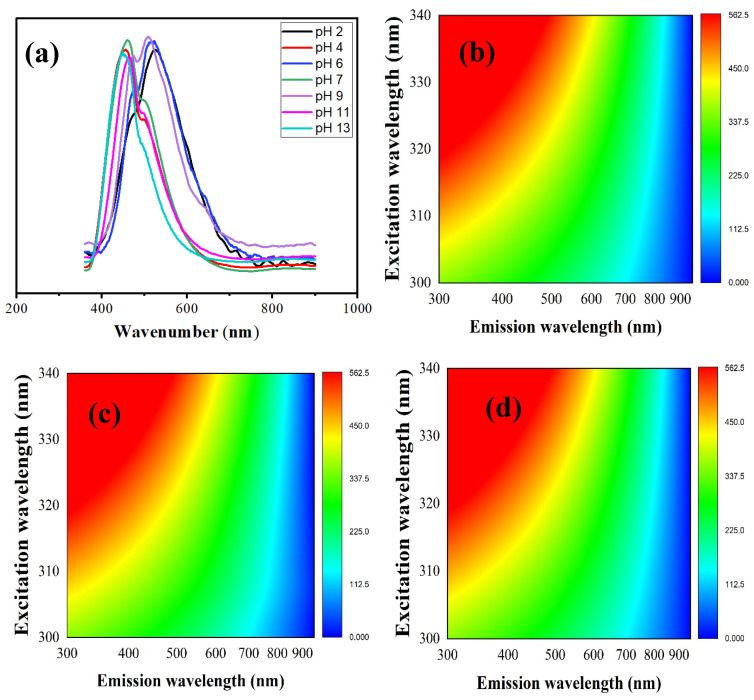

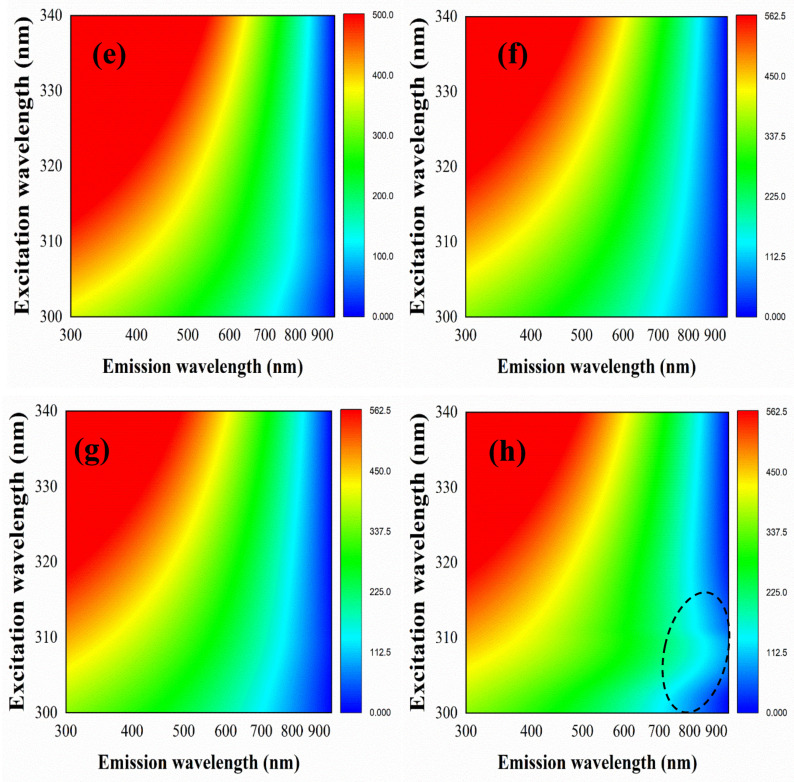
(**a**) Fluorescent spectra for Betalains-N–CQDs at different pH, (**b**) Color mapping at pH 2, (**c**) Color mapping at pH 4, (**d**) Color mapping at pH 6, (**e**) Color mapping at pH 7, (**f**) Color mapping at pH 9, (**g**) Color mapping at pH 11, and (**h**) Color mapping at pH 13 with a dotted circle show the change in the shape of the fluorescence.

To investigate the optical properties of the prepared Betalains-N–CQD sensor, we analyzed their fluorescence emission spectra at various pH levels, as shown in Figure 1a. When excited at 350 nm, the Betalains-N–CQDs consistently emitted light across the entire pH range of 2–13. These primary emission peaks, observed at wavelengths such as 471, 453, 473, 459, 475, 463, and 449 nm, are attributed to the inherent luminescence from the C=O and C=N moieties present within the Betalains-N–CQD structure. A notable observation was an additional emission peak in the range of 494–524 nm (specifically at 524, 501, 515, 503, 509, and 500 nm), which was present for pH values between 2 and 11. This longer-wavelength emission is ascribed to oxygen vacancy defects within the Betalains-N–CQDs [74]. However, a crucial change occurred at pH 13: this characteristic peak completely disappeared. This diminishment is primarily due to the high concentration of hydroxyl ions (OH^−^) in the extremely alkaline environment. These abundant OH^−^ ions act as effective quenching agents or passivators for the surface defects, including the oxygen vacancies. By strongly interacting with and effectively “filling” or “neutralizing” these defect sites, the OH^−^ ions reduce the non-radiative recombination pathways that normally contribute to the oxygen vacancy emission. Essentially, the defect state responsible for this particular luminescence is altered or eliminated by the strong interaction with the abundant OH^−^ ions, leading to a significantly diminished or quenched signal.

One primary mechanism involves the protonation and deprotonation of surface functional groups. Our Betalains-N–CQDs are rich in various surface groups, such as hydroxyl, carboxyl, amine, and carbonyl moieties, derived from their precursors and synthesis. These groups undergo varying degrees of protonation in acidic conditions and deprotonation in alkaline conditions. Even within a seemingly uniform acidic (e.g., pH 2, 4, 6) or alkaline (e.g., pH 9, 11) range, the incremental change in proton concentration at each pH unit causes a distinct shift in the protonation state of these groups. These changes in surface charge and electron density subtly alter the local polarity around the CQDs, which, in turn, influences the energy levels of both the surface states and the delocalized π–electron system. This perturbation can lead to minor shifts in the energy of electronic transitions, manifesting as slight red shifts or blue shifts in the observed peak wavelengths. For instance, the exact protonation state of hydroxyl groups near an oxygen vacancy defect could fine-tune the energy level of that defect state, causing a slight displacement in its associated emission. Beyond surface chemistry, changes in the local microenvironment and solvent relaxation also play a significant role. pH variations directly influence the hydration shell surrounding the CQDs and the local dielectric constant. This impacts solvent–solute interactions and the efficiency of solvent molecules reorienting around the excited fluorophores before emission. Different degrees of solvent relaxation can subtly alter the energy of the excited state, contributing to the small shifts in peak wavelength. Furthermore, while not explicitly detailed in our study, aggregation or disaggregation states are known factors for CQDs. Minor changes in surface charge at specific pH values can induce slight aggregation or disaggregation of the CQDs. Such alterations can lead to changes in inter–dot interactions or quantum confinement effects, and even subtle structural changes can influence the overall emission profile, including slight peak shifts. Finally, the inherent heterogeneity of CQDs, which possess multiple emission centers—including core luminescence from C=O/C=N moieties and various surface-state emissions from oxygen vacancies or other defects—contributes to these nuanced shifts. Each of these distinct emission centers may respond slightly differently to changes in pH. The observed fluorescence spectrum is, therefore, a convolution of these multiple emissions. Consequently, even if one dominant peak is shifting, the contributions of other, less intense, or overlapping emissions can influence the precise observed peak maximum, leading to the variations noted. In conclusion, these seemingly minor shifts within pH classifications are not random. Instead, they reflect the fine-tuning of the Betalains-N–CQDs’ electronic structure and local environment by incremental changes in proton concentration and the corresponding alterations in surface functional group states. This high sensitivity to subtle pH variations further validates the potential of our Betalains-N–CQDs as a precise and responsive pH sensor.

In addition to the emission spectra, Figure 1b–h present the fluorescence contour maps (FCMs), which provide a comprehensive visual representation of the Betalains-N–CQDs’ multicolor emission across the pH range of 2–13. These maps clearly illustrate how both the intensity and peak positions of the fluorescence change with varying excitation and emission wavelengths as a function of pH, highlighting the sensor’s highly responsive nature. A particularly interesting observation from these FCMs is the increase in blue color (representing emission in the blue wavelength region) at pH 13 (Figure 1h). This enhancement of blue emission, especially when considering the previous discussion of the diminished oxygen vacancy peak at this pH, is likely due to the dominance of the intrinsic C=O/C=N moieties’ emission. As the longer-wavelength emission attributed to oxygen vacancy defects is quenched at high alkalinity, the blue emission from the core electronic states of the carbon dots (primarily from the C=O/C=N groups) becomes more pronounced and visible. This shift in the dominant emission wavelength underscores the sensitivity of the Betalains-N–CQDs’ optical properties to extreme pH conditions, making them promising candidates for pH-responsive sensing applications.

### 3.2. DFT Calculations

**Figure 2:**
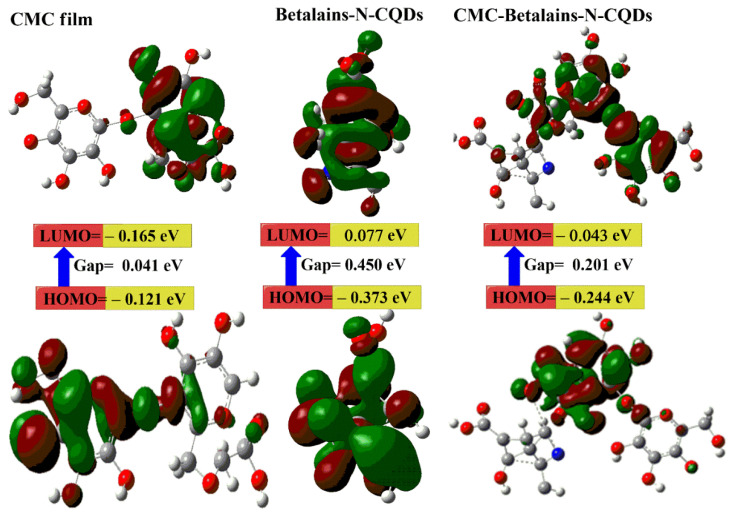
The gap energies (HOMO-LUMO) (eV) were calculated for the hydrogels using DFT B3LYP/6–31G (d), as was the molecular orbital interaction between CMC, Betalains-N–CQDs, and CMC-Betalains-N–CQDs.

DFT calculations were utilized to gain a deeper understanding of the stability and electronic characteristics of CMC, Betalains-N–CQDs, and their composite (CMC-Betalains-N–CQDs). The examination of Figure 2 and Table 1 provided crucial information regarding the molecular interactions and electronic structure of these materials. The seemingly counterintuitive result of the CMC-Betalains-N–CQD composite (4.96 Debye) exhibiting a lower μ than pristine CMC (11.14 Debye) arises from the intricate interplay of molecular interactions and structural arrangements within the composite material. While one might expect the incorporation of Betalains-N–CQDs to enhance polarity, the reality is that the individual dipole moments of the Betalains-N–CQDs and CMC are vector quantities. Their spatial orientation and the electronic interactions at the interface within the composite can lead to a partial cancellation of these moments. Furthermore, the introduction of Betalains-N–CQDs might induce conformational changes in the CMC chains, altering the orientation of their polar functional groups. The nitrogen doping within the CQDs themselves modifies their charge distribution, and their subsequent interaction with the CMC matrix can result in a composite structure with a more balanced or less asymmetric charge distribution overall compared to CMC alone. Consequently, this leads to a smaller net μ for the CMC-Betalains-N–CQD composite, indicating a lower overall polarity despite the addition of a potentially polar component. This offer has notable benefits for food preservation and safety. Primarily, a lower polarity enhances the material’s barrier properties against polar molecules such as water vapor and oxygen [75,76]. This is critical in food packaging as it minimizes moisture ingress that can lead to spoilage, microbial growth, and texture degradation, while also reducing oxygen permeation that can cause oxidation, flavor changes, and nutrient loss [77,78]. By creating a less polar barrier, the packaging effectively extends the shelf life and maintains the quality of the enclosed food product. Strawberries are highly susceptible to decay from excess moisture, which can encourage fungal growth [79]. A less polar barrier can help regulate the humidity levels within the package, preventing excessive condensation and maintaining an optimal environment for the fruit.

The observed low E_g_ for the CMC-Betalains-N–CQD composite (0.201 eV) compared to the Betalains-N–CQDs (0.450 eV) indicates a more stable and less reactive material. This stability can translate to enhanced barrier properties against the permeation of gases like oxygen and water vapor. Strawberries are susceptible to spoilage from both dehydration and oxidation [80]. Packaging with good barrier properties helps maintain the internal atmosphere, preventing moisture loss and limiting oxygen exposure, thus extending the shelf life and preserving the quality of the fruit [81,82]. In addition, the high E_g_ for the CMC-Betalains-N–CQDs compared to the pristine CMC proves that the CMC-Betalains-N–CQDs are less likely to interact with and allow the passage of metals or bacteria [83]. While a lower E_g_ is generally preferable for highly sensitive electronic or charge-transfer-based sensing of Pb(II) and bacteria, a composite with a higher E_g_ might still exhibit some sensing capabilities through alternative mechanisms. The sensitivity, selectivity, and detection limit would likely be different compared to a material with a lower E_g_. The Betalains-N–CQDs on the CMC matrix could still provide specific binding sites or surface interactions with Pb(II) or bacterial cells, even if the overall electronic transitions require more energy. These interactions might lead to detectable changes in other properties of the composite, such as its optical properties.

The increased stability and resistance to bond breakage in the CMC-Betalains-N–CQD composite can be partly attributed to its lower calculated E_T_ of −2006.20 au. This lower energy state suggests that the composite’s formation is energetically favorable, releasing energy in the process [43].

### 3.3. Shape Memory Test

**Figure 3:**
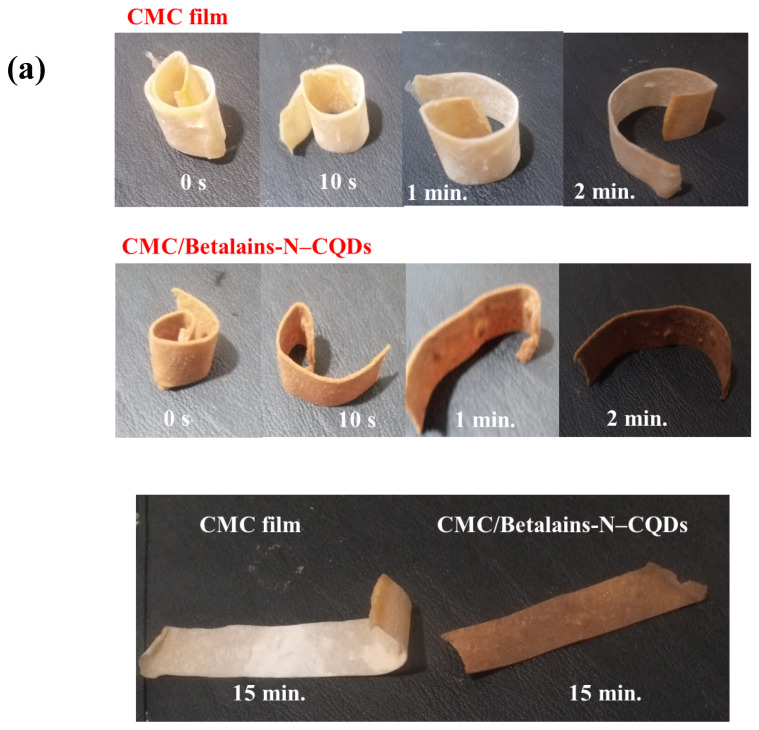

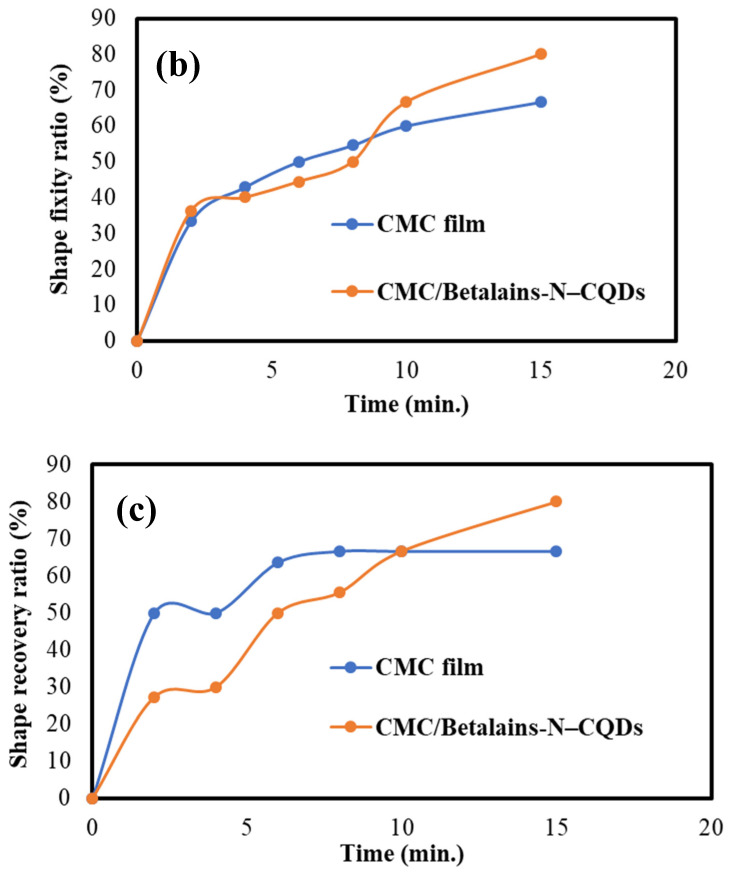
(**a**) Photographs of shape memory behavior of CMC film and CMC-Betalains-N–CQD films (**b**) Shape fixity ratios (R_f_) curve of CMC film and CMC-Betalains-N–CQD film with different times in alkaline solution (**c**) Shape recovery ratios (R_r_) curve of CMC film and CMC-Betalains-N–CQD films with different soaking times in acidic solution.

Figure 3a visually demonstrates the superior shape memory of the CMC-Betalains-N–CQD composite films compared to CMC films. When deformed into a spiral shape and then immersed in a 0.5 M HCl solution, the CMC-Betalains-N–CQD strip impressively returned to its original configuration within just 15 min. This rapid and complete recovery highlights its excellent shape memory capabilities. In contrast, the pure CMC film did not achieve a 100% restoration to its initial shape under the same conditions. This significant difference underscores the enhanced ability of the CMC-Betalains-N–CQD composite to “remember” and regain its original form, suggesting that the incorporation of Betalains-N–CQDs is crucial for these improved properties. These results indicate that the CMC-Betalains-N–CQD films can effectively hold various temporary shapes in alkaline conditions and reliably recover their original forms when exposed to an acidic environment.

Figure 3b provides a quantitative analysis of the Rf, which measures how well a temporary shape is maintained. For the CMC-Betalains-N–CQD composite films, the Rf steadily increased with time after soaking in the 0.5 M NaOH solution, reaching an impressive maximum of 80.00% after 15 min. This high value indicates that the CMC-Betalains-N–CQD composite film is highly effective at “fixing” or holding a deformed shape under alkaline conditions. Conversely, the CMC film exhibited a notably lower Rf of 66.66% for its temporary shapes. This direct comparison further confirms the superior shape fixity property of the CMC-Betalains-N–CQD film, demonstrating its enhanced ability to stabilize temporary deformations.

The non-covalent interactions form a robust yet reversible network within the CMC-Betalains-N–CQD composite. This dynamic network serves as the “memory” component, dictating the film’s original, permanent shape. When the film is mechanically deformed, for instance, into a spiral configuration, these weak, non-covalent interactions are temporarily disrupted or rearranged, allowing the material to hold the temporary shape. Upon exposure to the specific stimulus—in this case, the acidic environment—the crucial protonation of amine groups on the Betalains-N–CQDs is triggered. This protonation leads to the re-establishment of strong electrostatic interactions with other polar groups within the CMC matrix. Simultaneously, hydrogen bonds reform between the hydroxyl and amine groups, and π–π stacking interactions between the aromatic rings within the N–CQDs are re-aligned. This reversible breaking and reforming of these specific intermolecular interactions is the fundamental mechanism that enables the composite to recover its original shape rapidly and completely. These findings conclusively demonstrate that the temporarily deformed CMC-Betalains-N–CQD films can indeed recover their original shapes with high efficiency. This remarkable shape memory behavior is attributed to the intricate and dynamic interactions occurring between the Betalains-N–CQDs and the CMC polymer matrix. Specifically, this mechanism is mediated by three types of intermolecular forces: electrostatic attractions, hydrogen bonding, and π–π stacking. These interactions create a robust and reversible network that dictates the CMC-Betalains-N–CQDs’ ability to “remember” their original form. Under acidic conditions (such as immersion in 0.5 M HCl solution), the amine groups present in the Betalains-N–CQDs become protonated. These positively charged amine groups can then form strong electrostatic interactions with other polar groups in the CMC. Furthermore, hydrogen bonding between the hydroxyl and amine groups, found in both the CMC and Betalains-N–CQD components, respectively, further reinforces this network. Adding to this stability are the π–π stacking interactions that occur between the aromatic rings within the Betalains-N–CQDs, significantly enhancing the overall stability of the composite structure. When the CMC-Betalains-N–CQD film is mechanically deformed (e.g., bent into a U-shape), these vital interactions are temporarily disrupted. However, upon removal of the external stress and exposure to the appropriate stimulus—in this case, the acidic environment that triggers recovery—these specific interactions are able to reform. This reversible breaking and reforming of interactions is the fundamental mechanism that enables the CMC-Betalains-N–CQDs composite to recover its original shape, underpinning its superior shape memory performance when compared to CMC films. This sophisticated molecular interplay is the bedrock of its smart material properties. This exceptional shape memory behavior of CMC-Betalains-N–CQD films holds significant promise for food packaging applications, particularly for delicate fruits like strawberries. Strawberries are highly perishable and extremely prone to physical damage, such as bruising and compression, during handling, transportation, and storage. Traditional rigid packaging often fails to adequately protect them, leading to shortened shelf life and reduced market value.

### 3.4. Fourier Transform Infrared Spectroscopy (FTIR) Spectra and Morphological Observations

**Figure 4:**
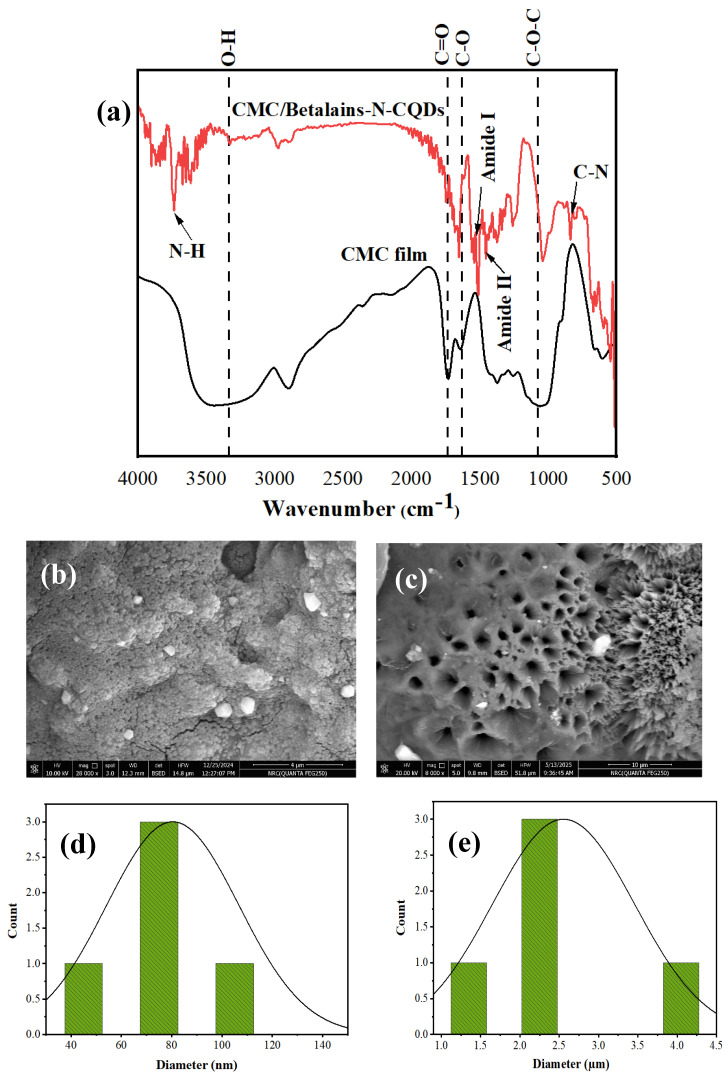
(**a**) FTIR spectra of CMC film and CMC-Betalains-N–CQDs; SEM analysis for (**b**) CMC film and (**c**) CMC-Betalains-N–CQDs; and pore size distribution for (**d**) CMC film and (**e**) CMC-Betalains-N–CQDs.

The CMC film and CMC-Betalains-N–CQDs showed peaks between 3525–3338 cm^−1^ (O–H), 1732–1747 cm^−1^ (C=O), 1641–1652 cm^−1^ (C–O bending), and 1036–1071 cm^−1^ (C–O–C pyranose ring). For CMC-Betalains-N–CQDs, the additional peaks at 3737 cm^−1^ (N–H), 1517 cm^−1^ (amide I), 1454 cm^−1^ (amide II), and 8438 cm^−1^ (C–N) prove the incorporation of Betalains-N–CQDs within the CMC film [28]. The O–H group of CMC film (3525 cm^−1^) was shifted to 3338 cm^−1^ for CMC-Betalains-N–CQDs, which means high H-bond strength (Figure 4a). The CMC-Betalains-N–CQD film’s larger pores, which range from 1.62 to 4.05 µm (Figure 4c), provide clear benefits, especially when it comes to the detection of larger analytes like bacteria and possibly some types of Pb(II) [36,73]. These larger channels offer an unhindered path for bacteria to effectively access the sensing elements embedded within the composite film, in contrast to the 48.18–119.20 nm pores of the pure CMC film (Figure 4b), which may impede the entry and interaction of micrometer-sized targets. In complex biological samples, such as strawberry environments, where the ability to freely enter and interact with the sensor material directly translates to improved sensing kinetics and signal generation, this increased accessibility is essential for achieving quick and reliable microorganism detection. Additionally, the CMC-Betalains-N–CQD film’s extensive pore network is very advantageous. For example, strawberry environments are high in proteins, sugars, and other particles that can quickly block nanoscale pores, reducing the lifespan and performance of sensors. Because of their size, the micrometer-scale pores provide a much lower tendency for this kind of entrapment, enabling the sample to flow freely and the target analytes to have continuous access to the active sites. For the development of useful and dependable sensors for on-site applications, this intrinsic resistance to fouling is essential since it reduces the need for intensive sample pre-treatment and increases the device’s operational lifespan.

### 3.5. Antibacterial Activity and Molecular Docking Study

Microbial contamination is a major factor in the post-harvest spoilage of strawberries, with studies identifying Escherichia coli, Staphylococcus aureus, and Candida as key organisms responsible [84,85]. Our results demonstrated that pristine CMC film, labeled as BB, lacked discernible antibacterial activity against Escherichia coli, Staphylococcus aureus, and Candida. Conversely, the CMC-Betalains-N–CQD composite, denoted as BT, exhibited significant antibacterial efficacy against these three species, evidenced by inhibition zones of 20, 21, and 21 mm, respectively. This enhanced antibacterial performance of CMC-Betalains-N–CQDs can be attributed to the inherent properties of the incorporated Betalains-N–CQDs, which facilitate multifaceted interactions with essential bacterial components. Specifically, the Betalains-N–CQDs, with their unique surface chemistry and electronic properties arising from nitrogen doping, can effectively interact with bacterial lipids, proteins, and nucleic acids through various bonding mechanisms. These include hydrogen bonding via surface functional groups, π–π stacking interactions with aromatic moieties in biomolecules, and electrostatic interactions arising from charged surface states. Such interactions disrupt crucial cellular processes, compromising bacterial membrane integrity, interfering with protein function, and hindering nucleic acid replication, ultimately leading to bacterial growth inhibition and cell death [36].

**Figure 5:**
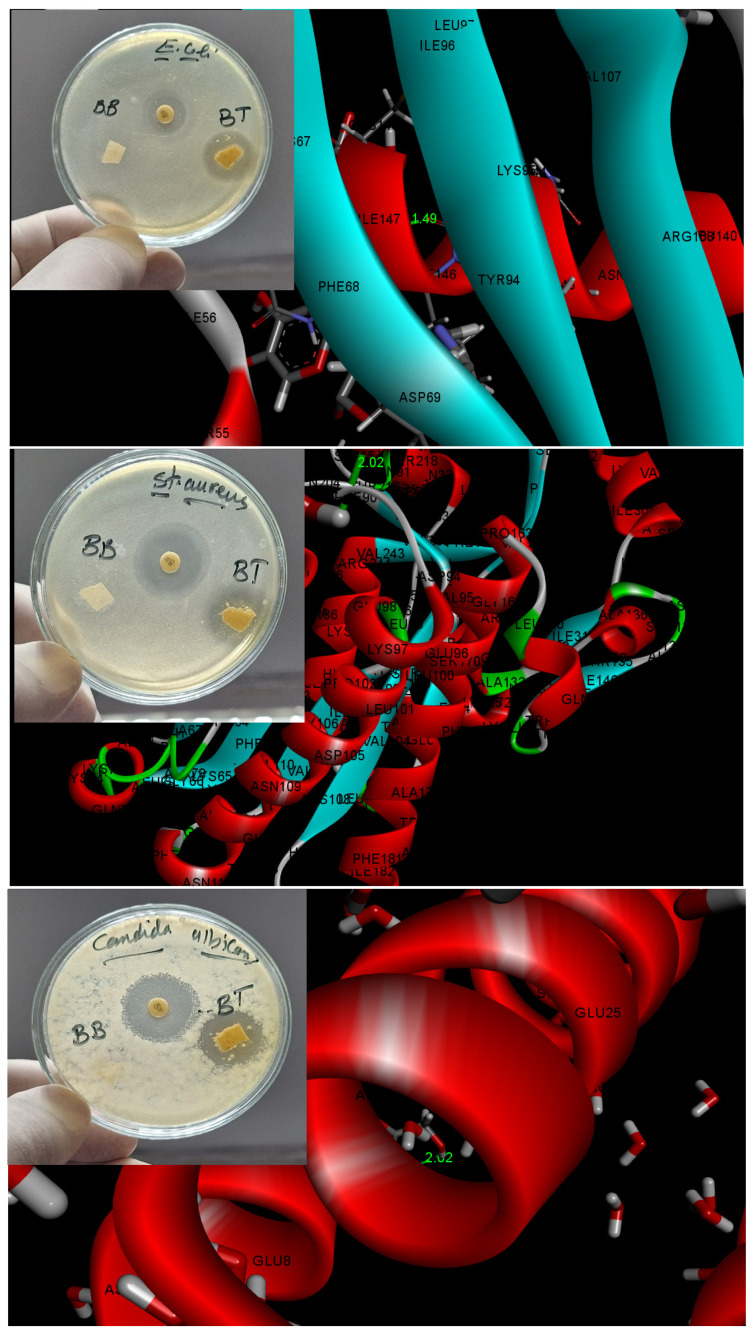
Antimicrobial activity of the CMC film (denoted as BB) and CMC-Betalains-N–CQDs (denoted as BT), and molecular docking study of CMC-Betalains-N–CQDs towards *Escherichia coli* PDB (3ZH7), *Staphylococcus aureus* PDB (7O39), and *Candida Albicans* PDB (6KWS) as receptor.

Figure 5 visually represents the analysis of the CMC-Betalains-N–CQD composite’s biological activity as a ligand interacting with specific protein receptors from *Escherichia coli* (PDB: 3ZH7), *Staphylococcus aureus* (PDB: 7O39), and *Candida albicans* (PDB: 6KWS). Molecular docking simulations revealed binding interactions between the CMC-Betalains-N–CQD ligand and the protein targets of *Escherichia coli*, *Staphylococcus aureus*, and *Candida albicans*, with predicted bond lengths of approximately 1.49 Å, 2.02 Å, and 2.02 Å, respectively. These computational findings corroborate our experimental observations regarding the antimicrobial efficacy of the CMC-Betalains-N–CQD composite. The notably shorter predicted ligand–protein bond length in the case of Escherichia coli suggests a higher degree of ligand reactivity and potentially stronger chelation or interaction with essential bacterial proteins. This strong interaction is likely to induce significant protein dysfunction, thereby contributing to the observed microbial growth inhibition and cell death [74].

### 3.6. CMC-Betalains-N–CQD Film as a Probe for Imaging Lead, Fungi, Bacteria

**Figure 6:**
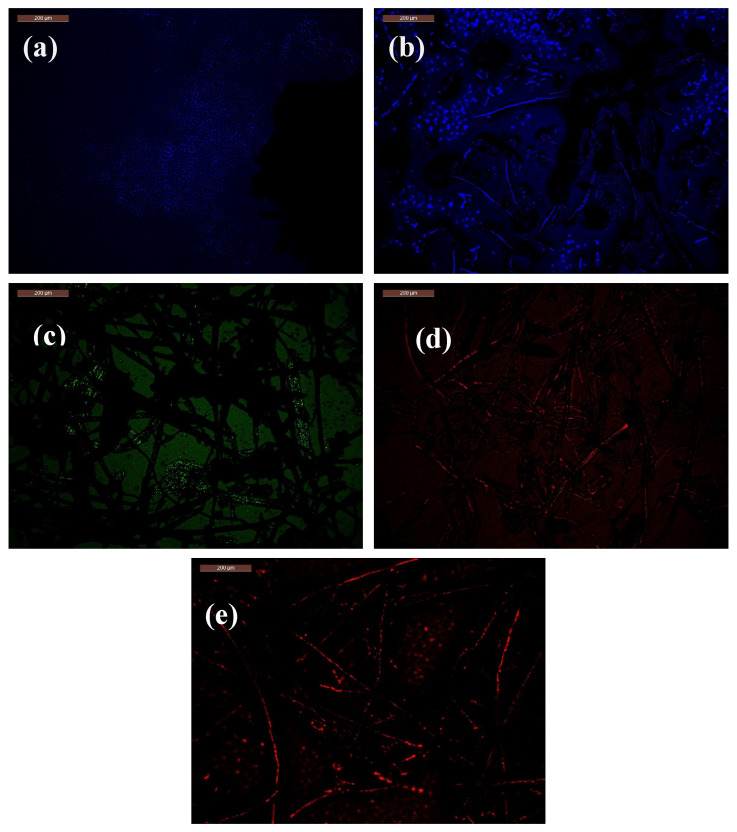
Fluorescence microscope for (**a**) CMC-Betalains-N–CQDs before bacterial contact, (**b**) CMC-Betalains-N–CQDs after contact with *Escherichia coli*, (**c**) CMC-Betalains-N–CQDs after contact with *Staphylococcus aureus*, (**d**) CMC-Betalains-N–CQDs after contact with *Candida Albicans*, and (**e**) CMC-Betalains-N–CQDs after contact with Pb(II).

The CMC-Betalains-N–CQDs were developed as a fluorescent sensor, exhibiting a distinctive blue fluorescence prior to any microbial interaction (Figure 6a), confirming their inherent luminescent properties. To assess their efficacy in microbial detection, we selected three common pathogens: The Gram-negative bacterium *Escherichia coli*, the Gram-positive bacterium *Staphylococcus aureus*, and the yeast *Candida albicans*. These pathogens were harvested during their exponential growth phase for subsequent confocal microscopy studies. After incubation with the CMC-Betalains-N–CQDs, the resulting cell-conjugates were thoroughly washed with double-distilled water and then imaged using a fluorescence microscope. This process revealed distinct and pathogen-specific fluorescence patterns upon interaction with each microorganism.

Upon exposure to *Escherichia coli*, the blue fluorescence of the CMC-Betalains-N–CQDs underwent a notable transformation, reorganizing into a wider blue sheet emission profile (Figure 6b). This unique response is attributed to the presence of *E. coli*’s lipopolysaccharide (LPS)-rich outer membrane. This outer membrane acts as a barrier, preventing direct and intimate interactions between the N–CQDs and the bacterial cell wall. The resulting LPS-mediated steric hindrance significantly alters the local environment and electronic interactions, consequently modifying the fluorescence emission to a wider blue wavelength [2]. When incubated with *Staphylococcus aureus*, the CMC-Betalains-N–CQDs produced a distinct green fluorescent sheet (Figure 6c). This difference in fluorescence profile, compared to *E. coli*, arises from the chemical composition of the *S. aureus* cell surface. Specifically, S. aureus possesses a higher concentration of negatively charged teichoic acids on its cell surface, a feature characteristic of Gram-positive bacteria, which interacts differently with the Betalains-N–CQDs than the LPS of Gram-negative bacteria [62,78]. In the presence of *Candida albicans*, the CMC-Betalains-N–CQDs exhibited a remarkable transition from blue to red fluorescence (Figure 6d). This observed color change provides strong evidence of the efficient internalization of the hydrophilic CMC-Betalains-N–CQDs into the *Candida albicans* cells. The oxygen and nitrogen functionalities present on the Betalains-N–CQDs facilitate this cellular uptake, likely through endocytosis, enabling the sensor to be incorporated within the fungal cells and subsequently alter its fluorescence properties.

The effectiveness of the CMC-Betalains-N–CQD film in detecting fungi and bacteria, as well as its colorimetric response to Pb(II) (fluorescence change color from blue to filamentous red), was evaluated in this study. This colorimetric response (blue to filamentous red upon Pb(II) interaction) indicates that the CMC-Betalains-N–CQD film could be effectively utilized as a visual sensor for monitoring Pb(II) levels within food packaging (Figure 6e).

### 3.7. CMC-Betalains-N–CQD Film as a Heavy Metal, Bacteria, Fungi and pH-Sensor for Tomatoes Spoilage by Naked Eye

**Figure 7:**
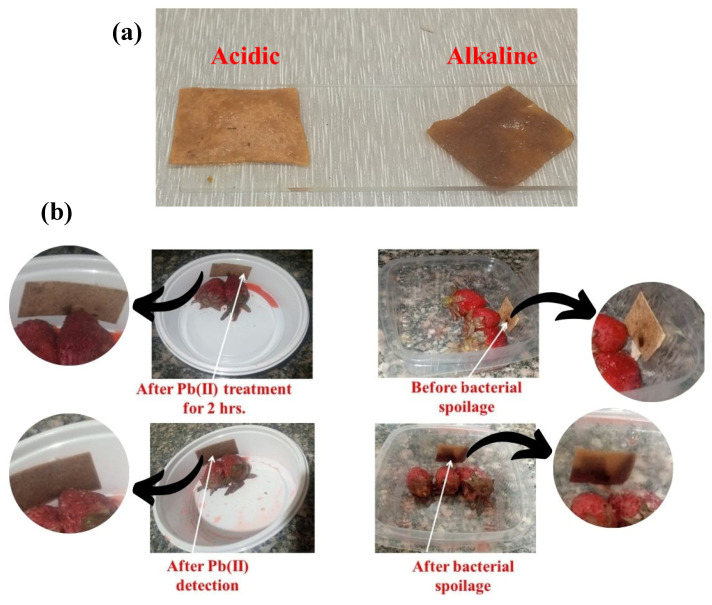
(**a**) Color response of CMC-Betalains-N-CQD film at acidic and alkaline media, and (**b**) Testing of CMC-Betalains-N-CQD on strawberry spoilage by bacteria and Pb(II).

The CMC-Betalains-N-CQD film, incorporating betalain-rich BR, exhibited a distinct color shift in response to varying pH conditions. Under alkaline conditions, the film displayed a brown color, while acidic solutions induced a yellow coloration (Figure 7a). The color shift observed is due to the inherent properties of betalains, the water-soluble pigments found in BR. However, under alkaline conditions, the structure of the betacyanins transforms. This leads to the appearance of yellow, as new betaxanthins are formed. This phenomenon demonstrates that CMC-Betalains-N–CQD films enriched with BR betalains are effective as pH-responsive indicators [86].

The CMC-Betalains-N-CQD film, while primarily designed for antimicrobial activity, may also indirectly influence pH dynamics through its interaction with bacterial and fungal metabolic products. The film’s interaction with the microbes and the strawberries themselves will create a complex system, and the overall pH of the strawberries will be the combination of these interactions. The conversion of strawberry sugars to organic acids during spoilage would also contribute to the overall pH shift. The CMC-Betalains-N-CQD film, by inhibiting microbial growth, aims to stabilize the pH and mitigate these spoilage-related pH changes. This resulted in strawberries stored with the CMC-Betalains-N-CQD film taking 7 days to spoil. Notably, after spoilage, the CMC-Betalains-N-CQD film exhibited a color transformation from its initial bright brown color to a more intense brown coloration, suggesting a chemical interaction between the film’s components and the byproducts of microbial degradation (Figure 7b; right side). Moreover, when Pb(II) polluted strawberries were stored with the CMC-Betalains-N-CQD film. The CMC-Betalains-N-CQD film underwent rapid colorization, transitioning from its initial bright brown color to an intense brown in a single day (Figure 7b; left side). This suggests a direct interaction between the Pb(II) within the film and the strawberries, leading to both the film’s color change.

## 4. Conclusions

This research successfully developed a sustainable, smart, and multifunctional intelligent packaging solution by incorporating betalains-nitrogen-doped carbon dots (Betalains-N–CQDs) derived from beet root waste (BR) into carboxymethyl cellulose (CMC) films derived from sugarcane bagasse agricultural waste. The resulting CMC-Betalains-N–CQD composite films demonstrated remarkable capabilities as a naked-eye sensor for strawberries, addressing critical concerns regarding food preservation and safety. Optical studies revealed the pH-responsive fluorescence of the Betalains-N–CQDs, with distinct emission patterns across various pH levels (2–13), highlighting their significant potential in sensing applications. The quenching of the oxygen vacancy emission at high alkalinity (pH 13) due to hydroxyl ion interaction and the subsequent dominance of blue emission from intrinsic C=O/C=N moieties underscored the sensitivity of these optical properties to extreme pH conditions. DFT calculations provided valuable insights into the composite’s stability and electronic characteristics, showing that the CMC-Betalains-N–CQD composite had a lower dipole moment and a higher band gap than pristine CMC. These properties are crucial for enhancing the material’s barrier properties against moisture and oxygen, directly benefiting the preservation of sensitive produce like strawberries by mitigating spoilage factors. Crucially, the CMC-Betalains-N–CQD film exhibited excellent shape memory capabilities, demonstrating the ability to fix various temporary shapes in alkaline conditions and reliably recover original forms in acidic environments. This behavior, attributed to dynamic electrostatic attractions, hydrogen bonding, and π–π stacking interactions, offers significant promise for the physical protection of delicate fruits, preventing bruising and maintaining structural integrity during handling and transport. Furthermore, the composite film displayed significant antibacterial activity against key foodborne pathogens, including *Escherichia coli*, *Staphylococcus aureus*, and *Candida albicans*, as evidenced by inhibition zones and corroborated by molecular docking simulations showing strong binding interactions with bacterial proteins. The film also functioned effectively as a fluorescent sensor, exhibiting distinct color changes upon contact with different microorganisms and Pb(II) heavy metals, enabling rapid, naked-eye detection. Beyond microbial and heavy metal detection, the film also acted as a pH sensor, displaying visible color shifts (brown in alkaline, yellow in acidic) due to the inherent betalains, proving useful for real-time monitoring of food spoilage in strawberries.

## Figures and Tables

**Table 1:** The quantum chemical parameters of CMC, Betalains-N–CQDs, and CMC-Betalains-N–CQDs.

**DFT B3LYP/6–31G (d)**	**CMC Film**	**Betalains-N–CQDs**	**CMC-Betalains-N–CQDs**
E _LUMO_ (eV)	−0.121	0.077	−0.043
E_HOMO_ (eV)	−0.165	−0.373	−0.244
E_g_ (eV)	0.041	0.450	0.201
E_T_ (au)	−1134.55	−244.29	−2006.20
μ (Debye)	11.14	2.98	4.96

## Data Availability

The original contributions presented in the study are included in the article; further inquiries can be directed to the author.
